# The Protective Effect of Silybin against Lasalocid Cytotoxic Exposure on Chicken and Rat Cell Lines

**DOI:** 10.1155/2013/783519

**Published:** 2012-12-30

**Authors:** Lidia Radko, Wojciech Cybulski, Wojciech Rzeski

**Affiliations:** ^1^Department of Pharmacology and Toxicology, National Veterinary Research Institute, Partyzantow 57, 24-100 Pulawy, Poland; ^2^Department of Virology and Immunology, Institute of Microbiology and Biotechnology, Maria Curie-Sklodowska University, Akademicka 19, 20-033 Lublin, Poland; ^3^Department of Toxicology, Institute of Agricultural Medicine, Jaczewskiego 2, 20-950 Lublin, Poland

## Abstract

Lasalocid, an ionophore coccidiostat, extensive use implies a risk of toxicological impacts. Protective effects of silybin, a herbal compound of *Silybum marianum*, are reported elsewhere. The aim of this study was to compare effects of the combined use of lasalocid and silybin in chicken hepatoma cells (LMH) and rat myoblasts (L6) cell lines cultures. The cytoprotective effect resulting from an interaction of both pharmaceuticals was measured with the help of MTT reduction and, coomassie brilliant blue binding (CBB) and LDH release assays. Isobolography and the combination index (CI) estimated the nature and scale of interaction. In all performed tests, the lowest lasalocid EC_50_-values were obtained for chicken hepatocytes. In the rat myoblasts cultures, the lowest lasalocid EC_50_-values were found with LDH test. Simultaneously, a lack of silybin cytotoxic effect was proven for the studied cell lines. An interaction between both substances led to a considerable decrease of lasalocid cytotoxicity. The isobolograms and combination index showed a significant antagonistic nature of silybin effect in the course of lasalocid cytotoxicity. It is concluded that the mechanism of cytoprotection results from complex reaction at biochemical and biophysical endpoints during chicken hepatocytes and rat myoblasts cell lines exposure to silybin and lasalocid co-action.

## 1. Introduction

Lasalocid is a polyether carboxylic ionophorous antibiotic intended for use in the veterinary practice as a coccidiostat for the gastrointestinal parasites' treatment in poultry. This drug is characterized by a narrow safety margin, since the accidental lasalocid poisoning cases among animals have been reported [1–4]. The clinical syndrome of lasalocid poisoning in animals includes skeletal muscle lesions, neurological signs, and increase serum enzymes,s which results from muscles and liver's damages [1–4]. The toxicological monitoring of eggs and chicken edible tissues (liver, muscles) revealed that the concentration of lasalocid is much higher than its maximum residue limits (MRL) [5, 6]. *In vitro *studies report that lasalocid disturbs the ions membrane transport, leading to mitochondria damages and cell functioning [7–9]. In herbal medicine, milk thistle (*Silybum marianum *L. Gaertn.) is a plant with a well-recognized cytoprotective activity due to silymarin content. The extract from fruits and seeds of milk thistle consists of silybin, as a main flavonolignan, representing 50% to 70% of the silymarin content [10, 11]. Both *in vitro *and *in vivo *studies reported its cytoprotective, antioxidant, and chemopreventive properties. The hepatoprotective effect of silybin has been studied in chicken intoxications induced by toxic agents, such as acetaminophen, carbon tetrachloride, iron overload, amanita mushroom poisoning, or aflatoxicosis [12–14]. Recently, silybin found application in the cardiomyo-protective treatment [15, 16]. Results of silybin study indicated that its activity is expressed as a cell membrane stabilizer and permeability regulator. In addition, promotion of ribosomal RNA synthesis, free radicals scavenging, and regulation of intracellular content of glutathione by silybin have been reported [17]. However, there is limited information regarding silybin interaction with veterinary drugs. 

The aim of this study is focused on lasalocid and silybin effects alone, followed by their combined simultaneous impact to liver and muscle cells, chicken hepatoma (LMH), and rat myoblast (L6) cell lines. Impact on the cell viability was examined by battery of tests, which evaluated the different endpoints at a cellular level. The MTT reduction to measure metabolism activity of living cells, coomassie brilliant blue dye (CBB) assay to evaluate total cellular protein, and lactate dehydrogenase release (LDH) to assess membrane stability tests were applied. The median effective concentrations (EC_50_) were estimated separately for lasalocid and silybin, followed by their combined effects measurement. Presentation of the data on isobolograms and estimation of combination index (CI) allowed to understand the nature of lasalocid and silybin interaction and its scale.

## 2. Materials and Methods

### 2.1. Chemicals and Reagents

The following chemicals were purchased from Sigma-Aldrich (St. Louis, MO, USA): silybin ≥98%, lasalocid sodium salt >97%, dimethyl sulfoxide (DMSO),   3-(4.5-dimethylthiazol-2-yl)-2.5-diphenyl tetrazolium bromide (MTT), coomassie brilliant blue R-250 dye, Trixon X-100, trypsin-EDTA, fetal bovine serum (FBS), antibiotic solution (penicillin and streptomycin), and L-glutamine. Dulbecco's modified Eagle media (DMEM) and Waymouth MB 751/1 were purchased from American Type Culture Collection (ATCC). All other chemicals were obtained from the commercial suppliers and were of the highest available purity.

### 2.2. Cell Cultures

Chicken hepatoma cell line (LMH) (ATCC) and rat myoblasts cell line (L6) (ATCC) were cultured in DMEM and Waymouth MB 751/1, respectively. The mediums were supplemented with foetal bovine serum (10%), antibiotics, and L-glutamine. Those cells were cultured in 75 cm^2^ cell culture flasks and kept at 5%  CO_2_, 95% air, at 37°C. The mediums were refreshed every 2 days and cells were trypsinized when they reached 70–80% confluence. The cells were counted using Bürker's hemacytometer. The initial cell viability was determined with the trypan blue exclusion test. The cell suspensions were placed into 96-well plates (NUNC) at a density of 2 × 10^5^ (LMH) and 2.5 × 10^5^ (L6) cells/mL in the incubation for 24 h. 

### 2.3. Exposure to Drugs

The concentration ranges (1–250 *μ*M) for lasalocid and silybin were selected on the basis of the results of the preliminary studies. The stock solution of lasalocid was prepared in DMSO, while silybin was prepared in ethanol. The final concentration of DMSO and ethanol was 0.1% in the medium. The same final concentrations of the solvents were used in the corresponding control. The effects of the substances, without the presence of cells, were measured as a blank. Each drug was tested in seven concentrations/six replications for 24 h. 

### 2.4. MTT Assay

The metabolic activity of living cells was assessed by the activity of dehydrogenases [18]. MTT was dissolved in a solution of sterile phosphate buffered saline at a concentration of 5 mg/mL and filtered through a 0.22 *μ*m filter to sterilize and protect from light. After incubation of the cells with the substances, 10 *μ*L of the MTT solution was added to each well of 96-well plates and incubated for 3 h at 37°C in humidified atmosphere of 5%  CO_2_. Formazan crystals were solubilised overnight in an SDS buffer (10% SDS in 0.01 N HCl), and the product was quantified at 570 nm wavelength. 

### 2.5. Coomassie Brilliant Blue (CBB) Assay

A total of cellular protein was measured by the coomassie brilliant blue R-250 dye uptake. Protein incorporation into the cells of the culture reflected the degree of cytotoxic effect of the studied substances [19]. The procedure was based on the INVITTOX Protocol no. 15 [20]. 

### 2.6. LDH Assay

The lactate dehydrogenase release was determined by means of commercially available Cytotoxicity Detection Kit, LDH (Roche Diagnostics, Poland). The assay was applied to measure membrane integrity as a function of the amount of cytoplasmic LDH released into the medium. Wells with cells and culture medium with 2% Trixon X-100 were the positive control [21]. 

### 2.7. Drug Interaction Analysis

The nature of the interaction between the studied drugs was analysed with the help of isobolography according to Chou and Talaly method, which is based on the estimation of cytotoxic median effect (EC_50_) [22–24]. 

The LMH and L6 cells were simultaneously incubated for 24 h with lasalocid at a median effective concentration (EC_50_) combined with silybin with the concentrations in the range from 1 to 250 *μ*M. Synergism or antagonism was depicted by the linear cell-kill effects obtained by the toxic drug in a combination with the interacting drug in the different concentrations [24]. The CI (combination index) mathematically compiled a two-drug pharmacological interaction and denominated its nature [22, 23]. 

### 2.8. Statistical Analysis

The obtained results (percentage of control values) are presented as mean values ± SD (standard deviation) of at least three independent experiments. Those data were assessed using one-way analysis of variance (ANOVA), then Dunnett *post hoc* test to determine the significance relative to an unexposed control. 

The concentrations necessary for 50% inhibition of viability of the cells for each substance (EC_50_) were calculated. The statistical comparisons among EC_50_ results were performed by an analysis of variance (ANOVA) followed by Tukey test. Differences were considered as statistically significant at *P* ≤ 0.05. 

## 3. Results

### 3.1. Effects of Lasalocid on Chicken Hepatoma Cells (LMH) and Rat Myoblasts (L6)

**Figure 1:**
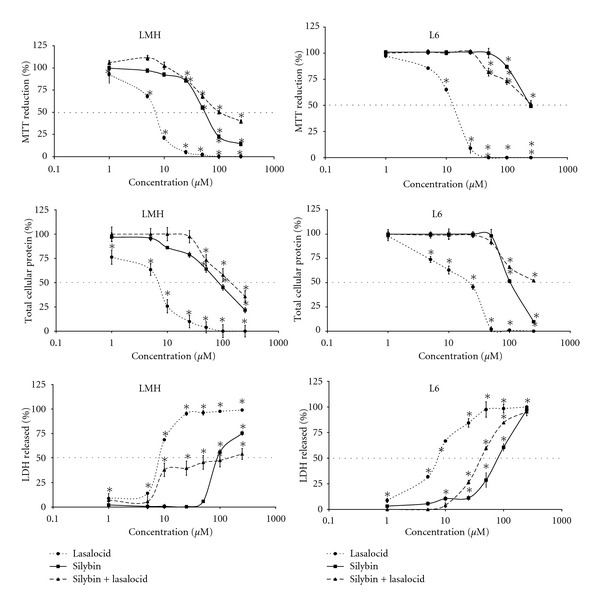
The effect of lasalocid, silybin, and silybin range concentrations in the interaction with lasalocid (EC_50_) on metabolism (MTT), total cellular protein and lactate dehydrogenase release (LDH) in chicken hepatoma (LMH), and rat myoblasts (L6) cell line. The values of three experiments are expressed as percentage of control response and are means ± SD (*n* = 3), *P < 0.05.

The cell metabolism, cellular protein content, and integrity of cell membrane were significantly affected in a concentration-dependent manner after 24 h exposure of chicken hepatocytes and rat myoblasts in the tested concentrations ranging from 1.0 to 250 *μ*M and compared to the control ones (*P* < 0.05) (Figure 1).

Lasalocid at concentrations above 10 *μ*M was cytotoxic for all chicken hepatocytes in performed three assays (Figure 1). 

Lasalocid at concentrations 1, 5, 10 *μ*M in rat myoblast cultures significantly (*P* < 0.05) affected the integrity of cell membrane, cellular protein content, and cell metabolism, respectively (Figure 1). 

The results of MTT, CBB, and LDH assays with lasalocid EC_50_ in LMH and L-6 cell lines are shown in Table 1. The mean values of effective concentrations of lasalocid in all assays with rat myoblasts were statistically different (*P* < 0.05), but it was not the case with chicken hepatocytes. The EC_50_ values of lasalocid on chicken hepatocytes were at least twofold lower than those values on rat myoblasts with the exception of LDH test, where the values for lasalocid were at the same level in both cell lines (Table 1). 

### 3.2. Effects of Silybin on Chicken Hepatoma Cells (LMH) and Rat Myoblasts (L6)

Silybin at a concentration higher than 25, 50, and 100 *μ*M decreased cellular metabolism, cellular protein content, and integrity of the cell membrane in chicken hepatocytes, respectively (*P* < 0.05) (Figure 1). 

Silybin at a concentration 100 *μ*M decreased metabolism and the cellular protein content in rat myoblasts. However, membrane integrity was affected at 10 *μ*M of the drug concentration; an increased LDH release was recorded at a significant level (Figure 1). 

The results of MTT, CBB, and LDH assays with silybin EC_50_ in chicken and rat cells are shown in Table 1. The EC_50_ values for silybin in both cell lines were of one to two orders higher when compared to lasalocid EC_50_ values. The EC_50_ from MTT assay was significantly (*P* < 0.05) lower than the values from EC_50_ for CBB and LDH tests carried out with chicken hepatocytes (Table 1). The EC_50_ values of MTT assay with silybin on chicken cells were threefold lower than that value for the rat myoblasts, whereas CBB and LDH tests yielded EC_50_ values at the same level on both cell lines (Table 1). The mean values of effective concentrations of silybin in MTT, CBB, and LDH assays with myoblasts were statistically different (*P* < 0.05). 

### 3.3. Effects Silybin on Cytotoxicity of Lasalocid on Chicken Hepatoma Cells (LMH) and Rat Myoblasts (L6)

Subsequent concentrations within 1–250 *μ*M range of silybin in co-action with lasalocid EC_50_ tested by MTT, CBB, and LDH assays stemmed for an estimation of marked interaction scale (Table 1, Figure 1). The values obtained for silybin in the interaction with lasalocid in MTT assay on chicken hepatocytes were significantly lower, than the EC_50_ estimated by use of CBB and LDH tests. The combined effect of both drugs yielded higher values than EC_50_ values obtained for silybin acting alone in chicken hepatocytes and rat myoblasts, with an exception of the results of LDH assay in the rat cells cultures (Table 1). The assays with rat myoblasts run for silybin in the interaction with lasalocid enabled to estimate EC_50_ value only at LDH test. 

### 3.4. The Nature of Interaction

**Figure 2:**
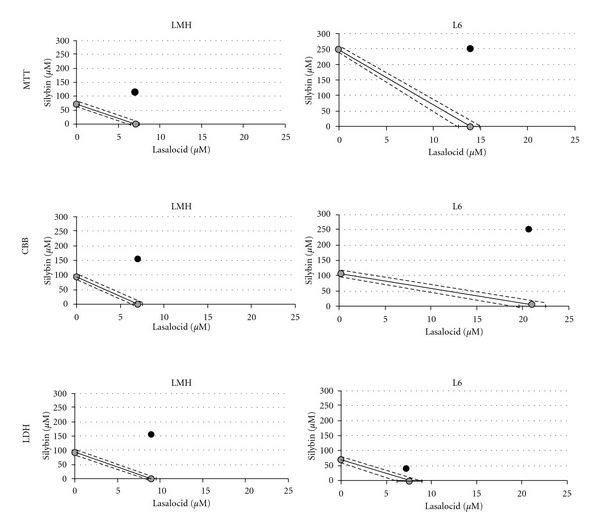
Isobolograms describing the interaction of silybin with lasalocid in LMH and L6 cells. The isobolograms were constructed by connecting the EC_50_ values of lasalocid with the EC_50_ of silybin. The black heavy lines indicate the theoretical line of additivity. The results below the additive line indicate synergism and those above the additive line denote antagonism.

**Figure 3:**
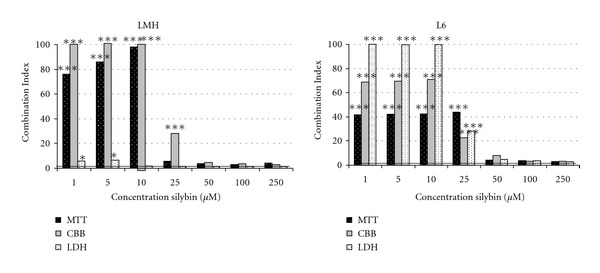
Values of thecombination index (CI) when lasalocid (EC_50_) was combined with silybin concentration range in LMH and L6 cells culture used conversion of tetrazolium salt (MTT) as an indicator of cell metabolism, coomassie brilliant blue (CBB) assay to evaluate cell proliferation and lactate dehydrogenase release (LDH) as an indicator of membrane integrity. CI value significantly higher than 1 indicates antagonism, CI not significantly different from 1 indicates addition, and CI significantly less than 1 indicates synergism (***P < 0.001).

The nature of the interaction between silybin and lasalocid in the chicken hepatocytes and rat myoblasts, analyzed with the help of isobolography, was depicted in Figure 2. It shows an antagonistic character of the interaction that affects the cell metabolism, cellular protein content, and membrane integrity of chicken hepatocyte and rat myoblast cell-lines. An extent of the antagonistic interaction was shown by CI > 1, values which were obtained from the fractional cell-kill levels (Fa) at both culture cells (Figure 3). The strong antagonistic effect of both compounds has been demonstrated for cellular metabolism and total protein contents in the hepatocytes. However, in the myoblasts the antagonistic effect of both interacting compounds was revealed for cellular membrane integrity and the total protein content. Antagonism was proven statistically, achieving combination index above 1 (CI > 1) at all tests assayed on chicken hepatocytes and rat myoblasts (Figure 3). The weak antagonism was noted at a higher concentration than 25 *μ*M of silybin coacting with lasalocid EC_50_ at both cell lines (Figure 3).

## 4. Discussion and Conclusion

The conducted studies represent a preliminary *in vitro* investigation aiming to quantify the potentials for the coccidiostat and silybin interaction. In this study, we used cell lines derived from chicken hepatoma (LMH) and rat myoblasts (L6). The cell line cultures do not display all abilities of primary cell cultures, as the last ones retain more of physiological and biochemical functions. Nevertheless, the cell line cultures represent some good characteristics enabling to estimate a nature and a scale of cytotoxic effects. The cells are homogeneous and of long viability. Moreover, they allow to avoid preliminary step with animals keeping along with 3R rules. The LMH cell line was shown to possess a liver-like enzyme pattern including the enzymes involved in biotransformation [25, 26]. 

 Lasalocid's median effective concentration was determined in chicken hepatoma cells in order to find out the extend of the inhibition of cellular metabolism, decrease of the cellular protein content, and disintegration of cell membrane, which served as the basic cytotoxicity biomarkers. The inhibition of cells viability was found at rather low concentration of lasalocid, which indicates on its potential cytotoxicity. Our findings are in accordance with other studies carried out on different cultures. The results both from primary cells and cell lines assays proved that lasalocid causes a chain of cellular damages, which lead to the death of cells [8, 27–29]. The studies on FaO and HepG2 cell lines showed clearly that lasalocid toxicity targeted cell metabolism [29, 30]. The EC_50_ of lasalocid MTT assay was found in range 4.0–9.0 *μ*M on rat (FaO) and human (HepG2) cell lines, which corresponds with EC50 for chicken cell lines (LMH). These results suggest similar sensitivity of those metabolizing cells to the coccidiostat. 

 The mechanism of lasalocid toxicity implies cytochrome P-450 microsomal enzymes involvement. Their catalytic activity can lead to formation of toxic metabolites, including the reactive and free radical derivatives of the coccidiostat [31]. Our results showed that chicken hepatocytes were more sensitive to the coccidiostat than rat myoblasts. It suggests that the role of lasalocid metabolites was involved in cytotoxicity. Myoblasts are the poor metabolizing cells and as a result EC_50_ values were up to two times higher than at biochemically active chicken hepatocytes. The lower lasalocid EC_50_ value of LDH test with myoblasts reflected the toxicant targeting towards a membrane as the most sensitive endpoints of cell damage (Table 1). The coccidiostat is responsible for changes in intracellular Ca^2+^ level. The formation of complexes with cations leads to mediate their transport across the cell membrane in response to diffusion gradients. Therefore, failures of ion pumps result in elevated intracellular sodium and calcium concentrations. Subsequently, mitochondrial swelling, metabolic disruption, and necrosis are developed [32]. 

 Detoxifying properties of the milk thistle (*Silybum marianum L.*) extracts are connected with silymarin, a mixture of flavonolignan compounds. Silybin, the major constituent of silymarin, is a chemically standardized substance, which enabled its application in numerous *in vivo *and *in vitro *studies. All of them indicated a lack of silybin toxicity [29, 33–35]. The EC_50_ for silybin was more than ten times higher than EC_50_ for lasalocid in all endpoints tested. The literature data show that concentrations of silybin up to 100 *μ*M and longer exposure than 24 h led to apparent inhibition of cells growth; however, no cytotoxic effects were found [34–37]. We showed that silybin in the concentrations lower than 100 *μ*M decreased cellular metabolism of chicken hepatocytes and increased the amount of LDH released from rat myoblasts. However, Chlopciková showed none cytotoxic effect of silybin on rat cardiomyocytes in concentration range 25–100 *μ*M and proved that the cardiomyocyte membranes are stabilized by silybin [16]. The mechanism of silybin effect may be explained by its hydrophobic character. This feature enables silybin penetration mainly to lipid bilayer component of the membrane leading to strengthen its biophysical structure. These effects on membrane stabilization may correspond to the cell protection and lack of serious side effects after the drug administration [38]. 

 We have not found studies in the literature addressing the interaction of silybin with ionophore antibiotics. An interaction analyzed by Chou and Talalay method provided an assessment of cytotoxicity scale affected by combined use of silybin and lasalocid. It provided also an estimation of greater, equal, or smaller effects of lasalocid in coaction with silybin than for their action alone. Our study showed a decrease of lasalocid cytotoxicity after its simultaneous exposure with silybin on both chicken hepatocytes and rat myoblasts. The data analyzed from Figure 1 and Table 1 proved a significant interaction extent between those substances both on the chicken hepatocytes and rat myoblasts, which reflected protective effect of silybin in course of lasalocid cytotoxicity. The EC_50_ values obtained from MTT, CBB, and LDH assays analyzed by isobolograms showed antagonism of lasalocid and silybin interaction, which resulted in protection of cells derived from chicken liver. Furthermore, the EC_50_ values from MTT and CBB assays with rat myoblasts were higher than the concentrations range of the drugs used in the assays with the hepatocytes. This implies different mechanisms of toxicity within the target cells and different antagonism scale between the studied drugs. Competitive bindings are probably involved in cytoprotective mechanisms of silybin and lasalocid interaction. If the components used as a mixture have different affinities towards the same binding site of the target cells, some of them could not be able to induce their toxicity. The antagonistic interactions were observed in cases of combination of silybin and phalloidin or ethanol [39]. The data of our previous studies have been shown an antagonistic interaction between lasalocid and silybin on rat (FaO) and human (HepG2) hepatoma cell lines. As a result of silybin interference a significant decrease of lasalocid cytotoxicity was revealed in both cell cultures [29, 30]. Hepatocellular injury due to high level of lasalocid seems to be the primary event. It is a consequence of the drug action itself; however; the toxic metabolites are responsible for cellular damages, too [40]. Silybin displays a regulatory effect on cellular membrane permeability in association with an increase of membrane stability. It prevents against xenobiotics injury, as control of a toxin absorption into the cells by occupying the binding sites as well as inhibiting many transport proteins in the membrane is involved. Considering the protective properties of silybin, we should take into account its antioxidant effect, which could be particularly visible by metabolism stimulation in chicken hepatocytes. Silybin reduced cytotoxicity induced by benzo(a)pyrene, bleomycin, and aflatoxin B_1_ [35]. Furthermore, silybin potentiated antitumor action of doxorubicin, cisplatin, carboplatin, and baicalein by synergistic effects leading to cell growth inhibition [37, 41]. The synergism or antagonism is probably an intricate balance between the concentration and type of antioxidants used, the class of chemotherapeutic agents administered, and the category of a cell involved [34]. 

 In general, it is difficult to clarify the full mechanisms underlying the effects of both drugs used in combination. It is possible that the interactions of silybin and lasalocid are because of some unknown mechanism related to complex perturbations of biochemical processes. 

Nevertheless, the obtained results suggest *in vivo *interaction study when simultaneous presence of lasalocid and silybin in feeding is used. Hence, investigation on target animals can pose a rationale of therapy safety margin assessment and lasalocid residue tissue formation. Both issues are relevant to protection of animal welfare and public health.

## Figures and Tables

**Table 1:** Effective concentration, EC_50_ (*μ*M) of lasalocid, silybin, and lasalocid EC_50_ in the interaction with silybin 1–250 *μ*M range concentrations estimated by the MTT, CBB, and LDH assays on chicken hepatoma (LMH) and rat myoblasts (L6) cell line, mean ± SEM, (*n* = 3).

	Lasalocid		Silybin		Silybin with lasalocid (~EC_50_)	
	LMH	L6	LMH	L6	LMH	L6
MTT	7.0 ± 0.54^a^	14.0 ± 1.08^b^	70.4 ± 1.05^ a^	247 ± 3.90^ c^	114 ± 11.37^ a^	N/D
CBB	6.8 ± 0.55^ a^	20.9 ± 1.36^c^	93.4 ± 2.63^ b^	104 ± 6.50^d^	154 ± 9.22^ b^	N/D
LDH	8.1 ± 0.54^ a^	7.6 ± 1.39^ a^	93.6 ± 0.98^ b^	70.5 ± 9.51^ a^	157 ± 6.65^ b^	42.7 ± 1.15^ c^

The different superscripts (a, b, c, d) within a column of compounds indicate significant differences between the methods (*P* < 0.05), N/D: not detected within the studied concentration range.

## Abbreviations

EC: Median effective concentration of drug
MTT: 3-[4.5-Dimethylthiazol-2yl]-2.5-diphenyl tetrazolium bromide
CBB: Coomassie brilliant blue
LDH: Lactate dehydrogenase
SD: Standard deviation
SEM: Standard error of the mean
ATCC: American Type Culture Collection.
